# Sudden cardiac death in HIV‐infected patients: A contemporary review

**DOI:** 10.1002/clc.23568

**Published:** 2021-02-16

**Authors:** Venkata A. Narla

**Affiliations:** ^1^ Section of Cardiac Electrophysiology, Division of Cardiology, Department of Medicine University of North Carolina at Chapel Hill Chapel Hill North Carolina USA

**Keywords:** HIV, sudden cardiac death, ventricular arrhythmias

## Abstract

HIV‐infected individuals have an increased risk of sudden cardiac death compared to the general population; yet the mechanisms underlying this increased risk remain unclear. The mechanisms underlying the heightened sudden cardiac death risk in HIV‐infected individuals is likely multifactorial. We reviewed the literature to elucidate and summarize the potential mechanisms contributing to sudden cardiac death in the HIV patient population. There is biologic plausibility that the following mechanisms may be contributing to the significantly heightened risk of sudden cardiac death in HIV to varying degrees: ventricular arrhythmias, myocardial fibrosis and scar, prolonged QTc interval (both as a direct effect of HIV on repolarization as well as a result of concurrent medications/antiretroviral therapies), substance abuse, structural heart disease, and premature atherosclerosis. Further understanding of the mechanisms underlying the increased sudden cardiac death risk in HIV can lead to identification of modifiable risk factors, implementation of public health programs, and potential revision of ICD implantation guidelines to ultimately reduce the incidence of sudden cardiac death in HIV‐infected patients. Further studies are needed to assess the relative contribution of each of these mechanisms and risk factors.

## 1 INTRODUCTION

**FIGURE 1:**
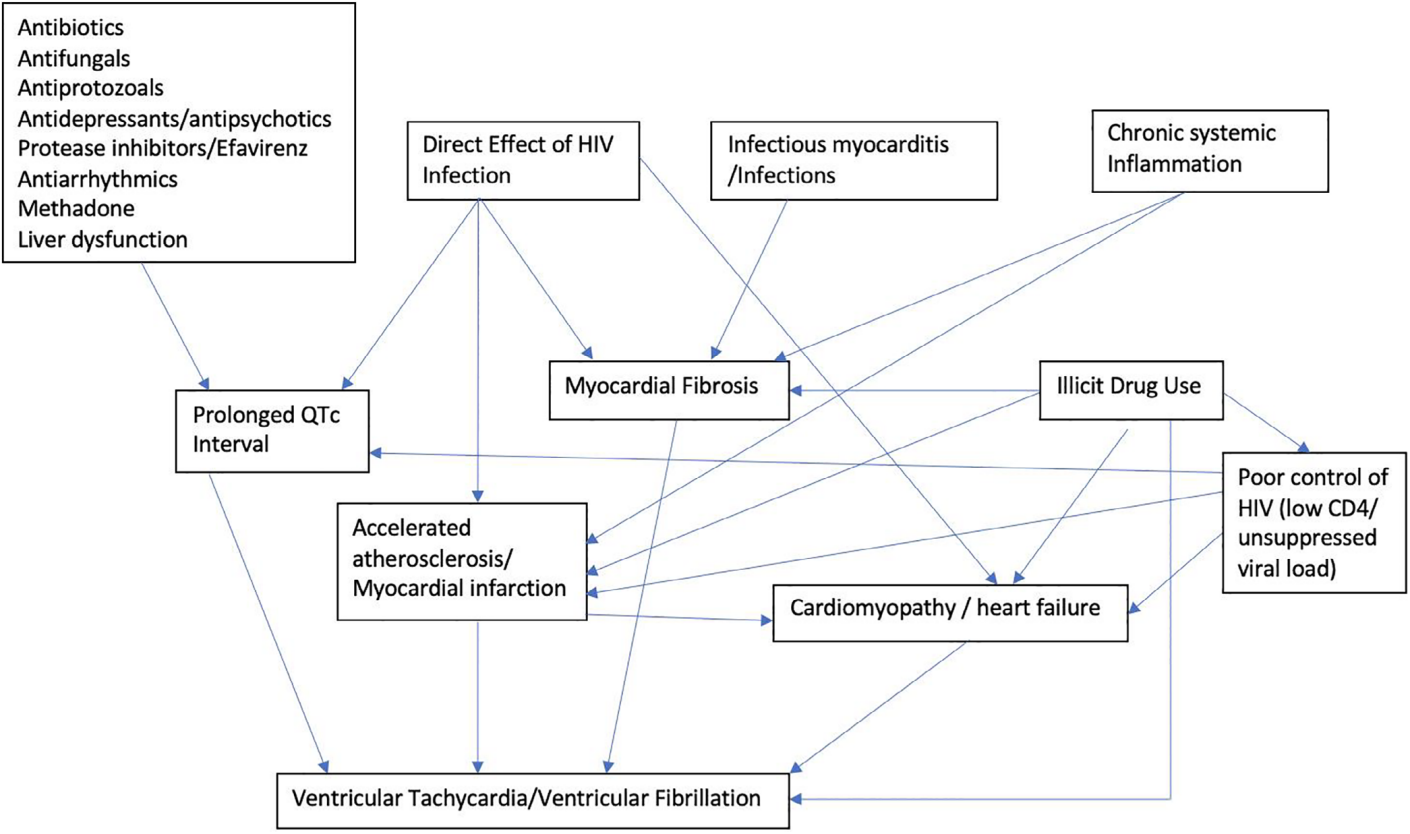
Complex interplay between possible mechanisms and risk factors for ventricular arrhythmias and sudden cardiac death in HV infection

HIV‐infected patients are now living longer due to the use of antiretroviral therapy (ART). As a result, chronic diseases such as cardiovascular disease (CVD) are becoming more prevalent in this particular patient population.^1^ HIV‐infected individuals have higher rates of CVD than uninfected individuals likely due to a combination of HIV‐associated inflammation, side effects of ART, and an increase in traditional risk factors among HIV‐infected individuals such as cigarette smoking, illicit drug use, hypertension, and dyslipidemia. HIV‐infected individuals have also been found to have up to a 4.5‐fold increased risk of sudden cardiac death (SCD) compared to uninfected individuals.^2^ This finding was further corroborated by Alvi et al^3^ in 2019 in a retrospective study of 344 HIV‐infected individuals hospitalized with heart failure as compared to 1805 uninfected controls hospitalized with heart failure at the same Medical Center. In this study, HIV‐infected patients had a 3‐fold increased risk of SCD compared to the uninfected controls (21% vs 6.4%, *p* < .001).^3^ After adjustment for various confounders, coronary artery disease, cocaine use, no use of beta blockers, low CD4 count/unsuppressed viral load, low left ventricular ejection fraction, increased QTc duration, and wider QRS were all independently associated with SCD among the HIV‐infected patients.^3^ The mechanisms underlying this increased risk of sudden cardiac death among the HIV‐infected population are not clearly understood but are likely multifactorial including ventricular arrhythmias due to chronic inflammation/myocardial fibrosis, increased rate of traditional cardiovascular risk factors, increased illicit drug use, side effects of antiretroviral therapy, prolonged QT interval, and accelerated atherosclerosis (Figure 1). We provide here a critical review of the current literature to elucidate and summarize the potential mechanisms contributing to sudden cardiac death in the HIV patient population, which in turn could be targeted for risk factor modification and prevention.

## 2 RISK OF VENTRICULAR ARRHYTHMIAS IN HIV‐INFECTED INDIVIDUALS RELATIVE TO UNINFECTED INDIVIDUALS

Alvi et al^4^ demonstrated in 2018 that among HIV‐infected subjects with concomitant heart failure, the rate of appropriate ICD discharges was higher than in uninfected controls. Additionally, ICD discharge was associated with increased cardiovascular mortality among both HIV‐infected and uninfected controls with heart failure.^4^ However, this study was limited to only subjects who had been admitted to the hospital for decompensated heart failure, which may represent a higher‐risk subgroup. Because appropriate ICD discharge is a surrogate for sudden arrhythmic death, this study nonetheless alludes to the higher risk of sudden arrhythmic death among HIV‐infected patients compared to uninfected subjects. On the contrary, Meyer et al^5^ demonstrated no significant difference in the prevalence of ventricular ectopy and ventricular tachycardia (VT) in a cohort of 4656 HIV‐infected individuals as compared to 5002 uninfected controls. However, a limitation of this study is the inherent heterogeneity of the outcomes of ventricular ectopy vs. VT, each of which portends a very different prognosis. Prevalence of VT alone was not compared in this study due to insufficient events. Nonetheless, among HIV‐infected subjects, poorer HIV control (as measured by nadir CD4 count and peak viral load) was associated with higher odds of ventricular ectopy/ventricular arrhythmias, thereby still potentially implicating ventricular arrhythmias in the possible mechanism of higher SCD risk among HIV‐infected individuals compared to the general population.^5^

## 3 MYOCARDIAL FIBROSIS

A higher prevalence of myocardial fibrosis has been noted in HIV‐infected individuals. Holloway et al^6^ found that among 90 HIV‐infected subjects on ART recruited from four HIV centers in the United Kingdom, HIV‐infected subjects were more likely to have myocardial fibrosis on cardiac magnetic resonance imaging (as assessed by T1 mapping and late gadolinium enhancement) as compared to matched uninfected controls (76% vs 13%, *p* < .001) . However, this study only included 39 matched uninfected controls and HIV‐infected subjects not on ART were excluded.^6^ They did not find an association between control of HIV (as measured by CD4 count/viral load) and degree of myocardial fibrosis. In a different cross‐sectional study of 95 HIV‐infected subjects and 30 matched uninfected controls with no known CVD, HIV‐infected subjects had greater indices of myocardial fibrosis as measured by cardiac MRI T1 mapping than the control group.^7^ The mechanism of development of myocardial fibrosis in HIV infection is unclear but possibilities include direct effect of HIV on the myocardium, side effects of ART, chronic systemic inflammation, and other infectious myocarditis. Similarly, myocardial fibrosis has been noted in other chronic inflammatory syndromes such as rheumatoid arthritis, a condition in which patients sometimes present with ventricular arrhythmias and ventricular ectopy of unclear origin.^8^ Larger prospective studies are needed to evaluate whether myocardial fibrosis is associated with a higher risk of scar‐mediated ventricular arrhythmias in HIV‐infected subjects and thus, a possible mechanism for the increased SCD risk in this particular patient population. This, in turn, could be a modifiable risk factor with ICD implantation and with potential implications for guideline modification.

## 4 QT PROLONGATION/RISK OF TORSADES DE POINTES

Numerous studies have reported an increased prevalence of QT prolongation among HIV‐infected individuals compared to uninfected controls possibly due to concurrent administration of QT‐prolonging medications as well as the effect of the virus itself on the QTc interval.^9^, ^10^, ^11^, ^12^ Table 1 summarizes six studies which compared the prevalence of QTc prolongation between HIV‐infected individuals vs. an uninfected control group.^9^, ^10^, ^13^, ^14^, ^15^, ^16^ QT prolongation is a precursor for life‐threatening arrhythmias including torsades de pointes. It is possible that there may be an inherent repolarization abnormality caused by HIV infection itself, which becomes more evident upon coadministration of QT prolonging drugs. For example, in an HIV transgenic mice model, a significant reduction in outward K+ currents was noted along with significantly prolonged QT interval compared to the wildtype uninfected control mice in the absence of any drug therapy.^17^ Heravi et al demonstrated in a cohort of 589 HIV‐infected individuals and 534 uninfected controls that HIV‐infected individuals had a higher QT interval variability, which is a marker of repolarization lability and has been found in other cohort studies to be predictive of SCD and ventricular arrhythmias.^18^, ^19^, ^20^

**TABLE 1:** Studies comparing prevalence of QTc prolongation between HIV‐infected subjects and uninfected controls

Author/year	Comparison groups (n)	Prevalence of QTc prolongation	Additional findings
Myerson et al. 2019^10^	HIV+ (156) vs HIV− (105)	29% vs 19%	In HIV+, ARV use was associated with lower odds of QTc prolongation (OR = 0.35, *p* = .04) and methadone use with higher odds of QTc prolongation (OR = 4.6, *p* = .01)
Sani et al. 2005^9^	AIDS pts (100) vs HIV+ asymptomatic (78) vs HIV− (80)	45% vs 28% vs 10%	
Ige et al. 2014^13^	Children aged 9 months to 14 years: HIV+ (100) vs HIV− (100)	18% vs 1%	
Ogunmodede et al. 2017^14^	HIV+ on ARV (76) HIV+ not on ARV (74)HIV− control (150)	17.3% vs 32% vs 4.7%	Mean QTc was longer among those with CD4 < 200 as compared to those with CD4 > 200 (0.445 ± 0.03 s vs 0.421 ± 0.03 s, *p* < .001). Prolonged QTc was more frequent in those with CD4 < 200 as compared to those with CD4 > 200 (50% vs 20.5%, *p* < .001).
Reinsch et al. 2017^15^	HIV+ males (413) HIV− male controls (826) HIV+ females (83) HIV− female controls (166)	22.8% vs 3.9% vs 12.1% vs 1.8%	After multivariable adjustment, smoking was independently associated with longer duration of QTc interval.
Njoku et al. 2016^16^	HIV+ on ARV (100) HIV+ not on ARV (100) HIV− controls (100)	18.2% vs 16.4% vs 10.5%	

Furthermore, HIV‐infected individuals are often treated with several other potentially QTc prolonging medications for complications of HIV. Patients are often treated with antibiotics (such as fluoroquinolones and macrolides), antifungals (such as voriconazole, fluconazole, and ketoconazole), antiprotozoals (such as IV pentamidine for treatment of pneumocystis jiroveci pneumonia), antipsychotics/antidepressants (such as haloperidol, seroquel, tricyclic antidepressants, and SSRIs), and antiarrhythmics (sotalol, dofetilide, and amiodarone), all of which are QT‐prolonging agents and may be synergistic with other risk factors in increasing the risk of torsades de pointes. There is an increased frequency of Methadone use for treatment of opioid abuse among HIV‐infected patients and methadone further increases risk of QTc prolongation in this patient population.^21^ Comorbid hepatic dysfunction such as from coinfection with hepatitis C can lead to decreased metabolism/decreased clearance of the above‐mentioned medications which can further prolong the QTc interval.^22^

Among ARTs, Protease inhibitors (PIs) have been implicated as potential QT prolonging agents by their dose‐dependent blockade of human Ether‐à‐go‐go‐Related Gene (HERG) potassium channels thereby increasing risk of torsades.^23^ However, some conflicting studies have demonstrated that PIs may not independently predispose to QT prolongation after adjustment for HIV and non‐HIV related risk factors .^24^, ^25^ When PIs are used with other QT prolonging medications or with a baseline prolonged QTc interval, cautious monitoring of QTc interval nonetheless is strongly advised. Efavirenz, a nonnucleoside reverse transcriptase inhibitor, has also been implicated in increased risk of QTc prolongation and torsades de pointes.^26^

## 5 SUBSTANCE ABUSE

The use of illicit drugs such as cocaine and amphetamines has been found to be significantly more common in HIV‐infected individuals than in the uninfected population.^3^, ^27^ It is well‐known that cocaine and/or amphetamines can lead to abrupt sympathetic surges, myocardial infarctions, and development of nonischemic cardiomyopathy, all of which can lead to increased risk of ventricular tachycardia and ventricular fibrillation. The mechanism of cocaine‐induced myocardial ischemia/infarction includes the following: increasing myocardial oxygen demand by elevating heart rate and blood pressure, inducing coronary vasoconstriction, activating platelets, increasing von Willebrand factor levels as well as fibrinogen levels, increasing risk of coronary aneurysms, and potentially leading to intracoronary thrombosis with or without plaque rupture.^28^ Ventricular arrhythmias that arise after cocaine use can be due to sodium channel blockade by the drug itself, a surge of sympathetic stimulation, and/or due to cocaine‐induced myocardial infarction.^28^ Alvi et al^4^ found that history of cocaine use was associated with greater likelihood of appropriate ICD discharge even after adjustment for history of coronary artery disease, CD4 count, use of beta blockers, QRS duration and higher NYHA class (*p* = .011). Furthermore, cocaine use has been found to not only lead to decreased ART adherence but also to directly affect HIV disease progression independent of adherence to ARVs.^29^ Therefore, given the multiple adverse cardiovascular effects of cocaine and amphetamines and given the documented high prevalence of illicit drug use in this patient population, the relative contribution of illicit drug use to the increased SCD risk in the HIV population may be quite high.

## 6 STRUCTURAL HEART DISEASE/ACCELERATED ATHEROSCLEROSIS

There is a high incidence of structural heart disease (especially diastolic dysfunction and pulmonary hypertension) among HIV‐infected individuals.^30^, ^31^ Increased and widespread use of ARTs has transformed HIV‐associated cardiomyopathy from a severe dilated cardiomyopathy to less severe systolic dysfunction and varying degrees of impaired diastolic dysfunction, independent of cardiovascular risk factors. Increased prevalence of cardiomyopathy and heart failure has been noted in HIV‐infected individuals who die from sudden cardiac death as compared to AIDS and natural deaths combined.^2^ Alvi et al^3^ found that the SCD rate was higher in HIV‐infected individuals hospitalized with heart failure compared to uninfected controls hospitalized with heart failure within all three of the LVEF strata that were investigated (<35%, 35–49%, >50%).

Furthermore, multiple studies have reported an increased prevalence of traditional cardiovascular risk factors, such as diabetes, HTN, and smoking among HIV‐infected individuals compared to the general population.^32^, ^33^, ^34^, ^35^ In addition to the traditional CVD risk factors, chronic systemic inflammation from viral infection contributes to endothelial dysfunction, immune activation, and vascular injury which further precipitates premature atherosclerosis in HIV‐infected individuals.^36^, ^37^, ^38^, ^39^, ^40^ HIV infection is associated with almost 50% increased risk of acute myocardial infarction as compared to the general population, even after adjustment for Framingham risk score, substance abuse, and comorbidities.^41^ This increased prevalence of structural heart disease and premature atherosclerosis in HIV‐infected individuals could be potential mechanistic etiologies for the increased sudden cardiac death risk in this patient population and potential substrate for ventricular arrhythmias; however, the relative contribution of each of these mechanisms remains unclear.

## 7 IMPLICATIONS FOR FUTURE RESEARCH

HIV‐infected patients who have an increased risk of SCD but do not meet current criteria for primary prevention ICD implantation may be missed opportunities for prevention of sudden death. There is biologic plausibility that the various aforementioned mechanisms of ventricular arrhythmias, myocardial fibrosis and scar, prolonged QTc interval (both as a direct effect of HIV on repolarization as well as a result of concurrent medications/ARTs), substance abuse, structural heart disease, and premature atherosclerosis all could be contributing to this significantly heightened risk of SCD. Further studies are needed to provide insight into the relative contribution of each of these mechanisms to the heightened SCD risk so that attention can be focused on the key modifiable risk factors. In particular, further studies are needed to assess the impact of myocardial fibrosis/scar on risk of ventricular arrhythmias in this patient population. Additionally, if further investigation corroborates that cocaine use may be one of the more important contributing factors to the increased SCD risk, more extensive and effective public health programs could be implemented for substance abuse counseling and assistance. Certain high‐risk patients could be identified for more aggressive risk factor modification with emphasis on blood pressure control, diabetes control, management of hyperlipidemia, and smoking cessation. In addition to these mechanisms, the impact of socioeconomic status/deprivation on the annual rate of HIV/AIDS mortality is quite substantial.^42^, ^43^ Lower socioeconomic status can lead to poorer access to healthcare and/or decreased adherence to ARTs.^44^ Poorer control of HIV (as measured by low CD4 count and unsuppressed viral load) has been independently associated with increased SCD risk in this patient population regardless of left ventricular ejection fraction.^3^ Such individuals may benefit from implementation of public health programs that improve access to coordinated high quality medical care. Further research studies are needed to identify this large proportion of vulnerable, high‐risk HIV‐infected individuals who do not fit the current standard guidelines for primary prevention ICD but nonetheless may benefit from modified guidelines for ICD implantation.

## Data Availability

Data sharing is not applicable to this article as no new data were created or analyzed in this study.
